# Multispectral imaging of T and B cells in murine tissue

**DOI:** 10.1186/2051-1426-3-S2-P256

**Published:** 2015-11-04

**Authors:** Zipei Feng, Mohammad Farhad, Bernard A Fox, Shawn Jensen

**Affiliations:** 1 Robert W. Franz Cancer Research Center, Earle A. Chiles Research Institute, Providence Cancer Center, Portland, Oregon, USA; 2 Earle A. Chiles Research Institute, Providence Cancer Center, Portland, OR, USA

## 

Assessment of patterns of immune infiltrates has been shown to be highly prognostic and diagnostic in many types of cancers. The clinical impact of such analysis is most notably shown in colon cancer, where objective quantification of CD3^+^ and CD8^+^ T cells densities in the primary tumor has a highly significant impact on patient prognosis. More recently, multiplex immunohistochemistry (IHC) has emerged as a powerful technique for the study of multiple immune parameters on a single slide. This not only increases efficiency, but more importantly, allows for the study of relationships between cell populations, offering greater insight into the mechanisms underlying various disease processes.

Currently, multiplex IHC platforms have been applied for use in human tissues, but its use in murine fixed-tissues is still being optimized. Many antibodies against human antigens are successfully used with paraffin sections for human IHC, but considerably less are available for mouse antigens. Notably, there has been a dilemma in the field about performing IHC staining on certain immune epitopes such as CD4 and CD8α, which stain functionally distinct T cell populations. These fixation sensitive epitopes are not detected in formalin-fixed paraffin embedded tissues, but have historically relied on frozen tissue for detection; however, this comes with sacrifices of the integrity of tissue architecture making it difficult to study tissue morphology. While it has been suggested that Zinc-based fixation buffers are superior in preserving these epitopes compared to formalin fixation, the specificity of the antibodies and the ability to multiplex under these conditions have not been tested. Being able to perform CD4 and CD8 staining reliably in paraffin-embedded murine tissues is critical to our understanding of their function in various physiological and disease processes. Especially given certain immune cell subtypes, such as tissue resident memory T cells, have been reported to be underestimated using standard flow cytometry techniques. In our study, we compared multiple fixation protocols as well as antigen retrieval methods to validate the use of multiplex IHC in murine tissues with sensitive epitopes such as CD4 and CD8α. Our approach allows for successful detection and quantification of 5 or more markers on murine tissues.

